# Refolding, crystallization and preliminary X-ray crystallographic study of the whole extracellular regions of nectins

**DOI:** 10.1107/S174430911100337X

**Published:** 2011-02-23

**Authors:** Hirotaka Narita, Atsushi Nakagawa, Yasunori Yamamoto, Toshiaki Sakisaka, Yoshimi Takai, Mamoru Suzuki

**Affiliations:** a Laboratory of Supramolecular Crystallography, Research Center for Structural and Functional Proteomics, Institute for Protein Research, Osaka University, Suita 565-0871, Japan; b Division of Membrane Dynamics, Department of Physiology and Cell Biology, Kobe University Graduate School of Medicine, Kobe 650-0017, Japan; c Division of Molecular and Cellular Biology, Department of Biochemistry and Molecular Biology, Kobe University Graduate School of Medicine, Kobe 650-0017, Japan

**Keywords:** cell-adhesion molecules, immunoglobulin-like domains, adherens junctions

## Abstract

The nectin family of Ca^2+^-independent immunoglobulin-like cell–cell adhesion molecules contains four members. Nectins, which have three Ig-like domains in their extracellular region, form cell–cell adherens junctions cooperatively with cadherins. The whole extracellular regions of nectin-1 (nectin-1-EC) and nectin-­2 (nectin-2-EC) were expressed in *Escherichia coli* as inclusion bodies, solubilized in 8 *M* urea and then refolded by rapid dilution into refolding solution. The refolded proteins were subsequently purified by three chromatographic steps and crystallized using the hanging-drop vapour-diffusion method. The nectin-1-EC crystals belonged to space group *P*2_1_3 and the nectin-2-EC crystals belonged to space group *P*6_1_22 or *P*6_5_22.

## Abstract 2

The whole extracellular regions of nectin-1 (nectin-1-EC) and nectin-2 (nectin-2-EC) were expressed in *E. coli* as inclusion bodies, solubilized in 8 *M* urea and then refolded by rapid dilution. Refolded nectin-1-EC and nectin-2-EC were subsequently purified using three chromatographic steps and crystallized by the hanging-drop vapour-diffusion method.

## 1. Introduction

Cell adhesion molecules are the primary mediators for various types of cell–cell junctions and play essential roles in various cellular processes, including morphogenesis, differentiation, proliferation and migration (Harris & Tepass, 2010 ▶; Ogita & Takai, 2006 ▶). In polarized epithelial cells, cell–cell junctions comprise several adhesive apparatuses including tight junctions (TJs), adherens junctions (AJs), desmosomes and gap junctions. AJs regulate TJ formation and the establishment of the apical–basal polarity at cell–cell adhesion sites (Takai, Ikeda *et al.*, 2008 ▶). Nectins, which are immunoglobulin-like cell adhesion molecules, and cadherins, which are Ca^2+^-dependent cell adhesion molecules, localize at AJs and have essential cooperative roles in AJ formation. Nectins initiate AJ formation before cadherins form cell–cell adhesions. Initial cell–cell contacts are formed between two neighbouring cells by nectins, and cadherins are then recruited to the nectin-based adhesion sites to form strong cell–cell adhesions. TJs are formed at the apical side of AJs. Together, nectins and cadherins mediate TJ formation by recruiting junctional adhesion molecules (JAMs), followed by claudins and occludins, to the apical side of AJs.

Nectins comprise a family of four members, nectin-1, nectin-2, nectin-3 and nectin-4, each with multiple isoforms (Takai *et al.*, 2003 ▶; Ogita *et al.*, 2010 ▶). Most nectin-family members are membrane glycoproteins with an extracellular N-terminal variable region-like domain, two extracellular constant region-like domains, a transmembrane region and a cytoplasmic tail. All nectins, except for nectin-1β, nectin-1γ, nectin-3γ and nectin-4, contain a PDZ-binding motif (E/A-*X*-Y-V) in their cytoplasmic tail. Through this sequence the nectins can bind afadin, which itself binds to an actin filament and α-­catenin.

Two nectin molecules on the surface of the same cell first form *cis*-­dimers, which is followed by the formation of *trans*-dimers of the *cis*-­dimers on apposing cells, resulting in the formation of cell–cell adhesions (Takai, Miyoshi *et al.*, 2008 ▶). Heterophilic *trans*-inter­actions have been detected between nectin-1 and nectin-3, between nectin-2 and nectin-3 and between nectin-1 and nectin-4. In addition to their cell adhesion activity, nectin-1 and nectin-2, but not nectin-3 or nectin-4, serve as entry receptors for α-herpesviruses by binding the virus-envelope glycoprotein gD with different specificities (Cocchi *et al.*, 1998 ▶; Sakisaka *et al.*, 2001 ▶; Lopez *et al.*, 2000 ▶; Geraghty *et al.*, 1998 ▶; Warner *et al.*, 1998 ▶). While nectin-1 shows activity as a receptor for herpes simplex virus type 1 (HSV-1), HSV-2, pseudo­rabies virus (PRV) and bovine herpesvirus type 1, nectin-2 mediates the entry of HSV-2 and PRV.

To further understand how nectin-family members form *cis*-interactions and *trans*-interactions, structural information on the extracellular region of nectin-family members is fundamental. Here, we report the refolding, crystallization and preliminary X-ray crystallo­graphic analyses of the extracellular regions of nectin-1 and nectin-2. To our knowledge, this is the first report of the crystallization of the whole extracellular regions of nectins.

## 2. Materials and methods

### 2.1. Protein expression and purification of inclusion bodies

Genes encoding the extracellular region of human nectin-1α (nectin-1-EC; residues 30–335) or mouse nectin-2α (nectin-2-EC; residues 32–339) were PCR-amplified using the Expand High Fidelity PCR system (Roche) from respective full-length cDNAs using primer A (5′-TA**GGATCC**GTCCCAGGCGTCCACTCC-3′; nectin-1α, forward) and primer B (5′-TA**GCGGCCGC**TTCTGTGATATTGACCTCCACC-3′; nectin-1α, reverse) or primer C (5′-TA**GGAT** **CCGC**AGGATGTGCGAGTTCAAGTGC-3′; nectin-2α, forward) and primer D (5′-TA**GCGGCCGC**GTCTCGCACCAGGATGACCT-3′; nectin-2α, reverse). The PCR products were inserted into the pGEM-T Easy vector (Promega), which contains 3′-T overhangs at the insertion site. Target genes were isolated by digestion of the plasmids with the restriction enzymes *Bam*HI and *Not*I and were confirmed by DNA sequencing. This was followed by ligation into the T7 promoter expression vector pET21b (Novagen) in frame with a C-­terminal 6×His tag. Each recombinant plasmid was transformed into *Escherichia coli* BL21 (DE3) cells (Novagen).

The cells were cultured in Luria–Bertani broth containing 100 µg ml^−1^ ampicillin at 310 K until the OD_600_ reached 0.5–0.7. Protein expression was induced at 298 K by the addition of isopropyl β-d-1-thiogalactopyranoside to a final concentration of 0.4 m*M*. At 16 h post-induction the cells were harvested by centrifugation, suspended in phosphate-buffered saline containing 40 µg ml^−1^ hen egg-white lysozyme, subjected to two cycles of freezing and thawing and sonicated until the lysate was homogeneous. Centrifugation of these lysates at 10 000*g* for 20 min yielded inclusion bodies.

The inclusion bodies were washed four times with a buffer con­sisting of 20 m*M* Tris–HCl pH 7.5, 300 m*M* NaCl, 1 m*M* EDTA, 0.5% Triton X-100 and 1 m*M* DTT and were dissolved in a buffer con­sisting of 50 m*M* MES–NaOH pH 6.0, 8 *M* urea, 1 m*M* EDTA and 1 m*M* DTT. This mixture was slowly rotated overnight at 277 K before centrifugation at 10 000*g* for 20 min to remove insoluble materials. The yields of inclusion bodies were ∼0.1 g for nectin-1-EC and ∼0.2 g for nectin-2-EC per litre of culture.

Unfolded proteins were refolded by 300-fold dilution into refolding solution *A* [500 m*M* l-arginine, 100 m*M* Tris–HCl pH 9.0, 2 m*M* oxidized glutathione (GSSG) and 1 m*M* reduced glutathione (GSH)] in the case of nectin-1-EC or refolding solution *B* (500 m*M* l-­arginine, 100 m*M* Tris–HCl pH 9.0, 10 m*M* GSSG, 0.1 m*M* GSH) in the case of nectin-2-EC, followed by incubation for 48 h at 277 K. After concentration using a 10 000 molecular-weight cutoff ultrafiltration membrane (GE Healthcare), the samples were sub­jected to size-exclusion chromatography on a HiLoad 16/60 Superdex 200 pg column (GE Healthcare) to separate correctly folded proteins from aggregated forms. These fractions were dialyzed against 20 m*M* MES pH 6.0 to precipitate almost-misfolded proteins, filtered using an Ultrafree-MC GV 0.22 µm (Millipore) and applied onto a HiTrap SP HP column (5 ml; GE Healthcare) followed by a Mono Q column (1 ml; GE Healthcare). The protein yields were ∼0.5 mg for nectin-1-EC and ∼10 mg for nectin-2-EC from 100 mg inclusion bodies.

### 2.2. Optimization of refolding conditions and protein purification

The standard conditions for refolding nectin-1-EC and nectin-2-EC were as follows: 400 m*M* l-arginine, 100 m*M* Tris–HCl pH 9.0, 2.5 m*M* GSH, 2.5 m*M* GSSG and 100 µg ml^−1^ unfolded protein. Small-scale refolding assays (1 ml) were performed to investigate the effects of changing the l-arginine concentration from 100 to 600 m*M* (for nectin-1-EC), the pH from 7.0 to 9.0 (for nectin-1-EC), the GSSG:GSH ratio from 10.0:0.1 m*M* to 0.1:10.0 m*M* (for both nectin-1-EC and nectin-2-EC) and the concentration of unfolded protein from 25 to 200 µg ml^−1^ (for nectin-1-EC). In each case, the unfolded protein solutions were diluted at least 300-fold into each of the refolding solutions such that only one parameter was varied while the other parameters were kept at the standard conditions.

**Figure 1:**
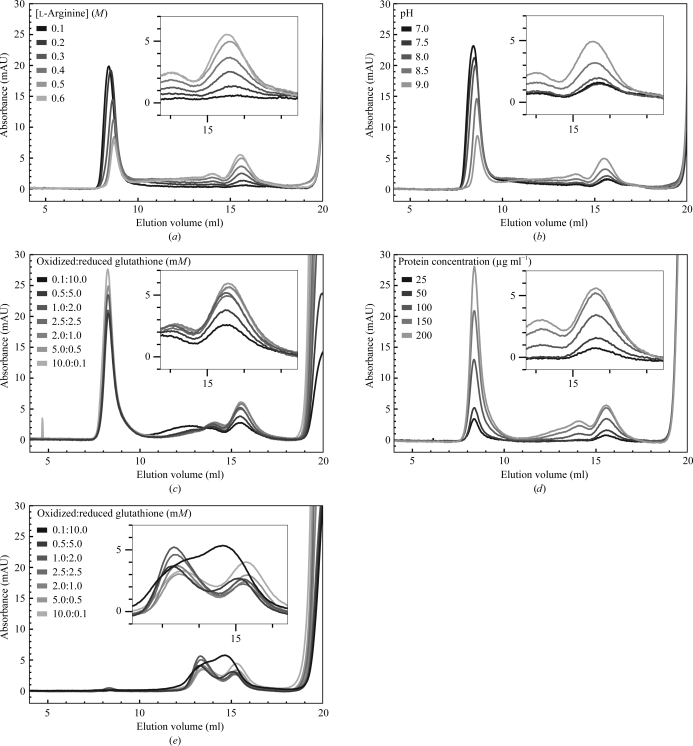
Optimization of refolding conditions for nectin-1-EC and nectin-2-EC. (*a*–*d*) Effects of varying (*a*) the l-arginine concentration, (*b*) the pH, (*c*) the GSSG:GSH ratio and (*d*) the protein concentration on the recovery of nectin-1-EC. (*e*) Effects of varying the GSSG:GSH ratio on the recovery of nectin-2-EC. Samples from small-scale refolding reactions were subjected to size-exclusion chromatography on a Superdex 200 10/300 GL column. Correctly folded proteins eluted between 15 and 16.5 ml. Aggregates eluted between 8 and 9.5 ml. The insets show close-up views of the elution profiles between 14 and 17 ml.

The solutions were incubated at 277 K for 48 h and then subjected to size-exclusion chromatography on a Superdex 200 10/300 GL column using an ÄKTA FPLC system (GE Healthcare; Figs. 1*a*–1*d* ▶). A peak at an elution volume of ∼15.5 ml corresponded to the correctly folded protein containing native intramolecular disulfide bonds. Aggregates containing intermolecular disulfide bonds eluted in the void volume (∼8.5 ml).

### 2.3. Crystallization

Purified nectin-1-EC and nectin-2-EC were dialyzed against solutions *C* (20 m*M* Tris–HCl pH 7.5, 150 m*M* NaCl) and *D* (20 m*M* Tris–HCl pH 9.0, 150 m*M* NaCl), respectively, and then concentrated to 5 and 4 mg ml^−1^, respectively, using a Vivaspin 6 10k (GE Healthcare). The homogeneous proteins were analyzed by screening additives using dynamic light scattering with a Zetasizer Nano ZS (Malvern Instruments) to determine their suitability for crystallization. In the presence of 0.2 *M* NDSB201 nectin-1-EC and nectin-2-EC were monodisperse. Initial crystallization trials were performed using a Phoenix liquid-handling system (Art Robbins Instruments) at 296 K using SaltRx 1, SaltRx 2, 50%(*v*/*v*) (half concentration) PEGRx 1, 50%(*v*/*v*) PEGRx 2, 50%(*v*/*v*) PEG/Ion 1 and 50%(*v*/*v*) PEG/Ion 2. The volume of the reservoir solution was 60 µl. The drops consisted of 0.2 µl of both the protein and reservoir solution.

**Figure 2:**
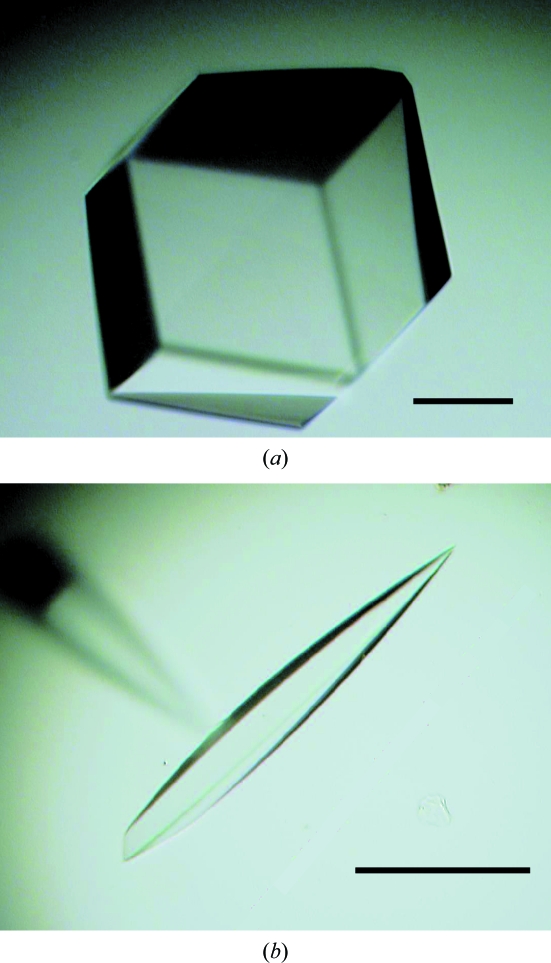
Crystals of the whole extracellular regions of nectins. (*a*) Nectin-1-EC crystal. (*b*) Nectin-2-EC crystal. The scale bars are 100 µm in length.

In the presence of 0.4 *M* NDSB201, nectin-1-EC and nectin-2-EC crystals were obtained using both polyethylene glycol and salt conditions as the reservoir. The initial crystallization conditions for nectin-1-EC and nectin-2-EC were further refined by changing the pH, precipitant concentration and additives. The most promising crystals of nectin-1-EC were observed in drops comprised of equal volumes of nectin-1-EC solution [20 m*M* Tris–HCl pH 7.5, 150 m*M* NaCl and 6%(*w*/*v*) 1,6-hexanediol] and precipitant solution [50 m*M* citric acid, 50 m*M* bis-Tris propane and 1–3%(*v*/*v*) PEG 3350] at 296 K (Fig. 2 ▶ *a*). Crystals of nectin-1-EC could be obtained even if the crystallization conditions contained no NDSB201, and the NDSB201 did not influence the diffraction quality of the crystals. The most promising crystals of nectin-2-EC were observed in drops comprised of equal volumes of nectin-2-EC solution (20 m*M* Tris-HCl pH 9.0, 150 m*M* NaCl and 0.35 *M* NDSD201) and precipitant solution (45 m*M* citric acid, 55 m*M* bis-Tris propane and 3.6 *M* sodium nitrate) at 296 K (Fig. 2 ▶ *b*).

### 2.4. Data collection

To improve the diffraction quality of the nectin-1-EC crystals, the crystals were subjected to dehydration with increasing concentrations of PEG 300. The crystals were transferred in a large number of steps from harvesting buffer [20 m*M* Tris–HCl pH 7.5, 150 m*M* NaCl, 6%(*w*/*v*) 1,6-hexanediol, 50 m*M* citric acid, 50 m*M* bis-Tris propane and 5%(*v*/*v*) PEG 3350] to harvesting buffer including 25%(*v*/*v*) PEG 300 at 277 K. Before freezing with liquid nitrogen, the crystals were equilibrated in the final buffer for 3 d. This procedure markedly improved the resolution limit of the crystals from ∼5 to ∼2.8 Å. The nectin-2-EC crystals were soaked in a cryoprotection solution consisting of 20 m*M* Tris–HCl pH 9.0, 150 m*M* NaCl, 0.4 *M* NDSB201, 45 m*M* citric acid, 55 m*M* bis-Tris propane, 4.0 *M* sodium nitrate and 14%(*v*/*v*) ethylene glycol by stepwise transfer at room temperature.

**Figure 3:**
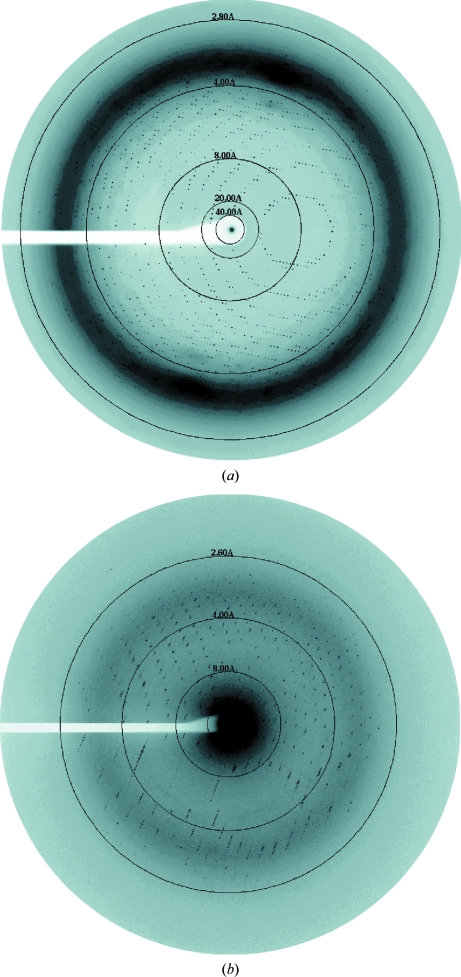
X-ray diffraction images from nectin crystals. (*a*) 0.5° oscillation image from a nectin-1-EC crystal. (*b*) 1.0° oscillation image from a nectin-2-EC crystal.

Diffraction data sets were collected from nectin-1-EC and nectin-2-EC crystals on the BL44XU beamline at the SPring-8 synchrotron facility (Harima, Hyogo, Japan) at 100 K using a DIP6040 imaging-plate detector (MAC Science/Bruker AXS). A total of 60 frames of data were collected for nectin-1-EC in three runs with a translation of 70 µm along the rotation axis. The nectin-1-EC data-collection parameters included a crystal-to-detector distance of 540 mm, an oscillation angle of 0.5° and an exposure time of 20 s per frame at a wavelength of 0.9000 Å (Fig. 3*a* ▶). For nectin-2-EC, a total of 70 frames of data were collected with a crystal-to-detector distance of 400 mm, an oscillation angle of 0.5° and an exposure time of 2 s per frame at a wavelength of 0.9000 Å (Fig. 3*b* ▶). All data sets were processed and scaled with the *HKL*-2000 program package (Otwinowski & Minor, 1997 ▶). Data-collection statistics are summarized in Table 1 ▶.

## 3. Results and discussion

The extracellular regions of nectin-1 and nectin-2 fused with a C-­terminal 6×His tag were expressed as inclusion bodies in *E. coli* BL21 (DE3). After solubilizing the inclusion bodies in 8 *M* urea, nectin-1-EC and nectin-2-EC proteins were successfully refolded by rapid dilution with a glutathione redox couple. To increase the yields of refolded nectin-1-EC protein, the refolding conditions (*i.e.* pH, GSSG:GSH ratio, l-arginine concentration and nectin-1-EC con­centration) were optimized in a series of small reactions (1 ml). Correct folding was assessed by size-exclusion chromatography on a Superdex 200 10/300 GL column using an ÄKTA FPLC system (GE Healthcare; Figs. 1*a*–1*d* ▶). Notably, a lack of the C-terminal 6×His tag significantly decreased the yield of correctly folded protein; we could not obtain crystals of nectin-1-EC without the tag. Based on the results for nectin-1-EC, only the GSSG:GSH ratio was optimized for nectin-2-EC (Fig. 2 ▶ *e*). The optimized refolding solutions for nectin-1-­EC and nectin-2-EC are described in §[Sec sec2]2. Nectin-1-EC crystals belonged to the cubic space group *P*2_1_3, with unit-cell parameters *a* = *b* = *c* = 164.9 Å. Nectin-2-EC crystals belonged to the hexagonal space group *P*6_1_22 or *P*6_5_22, with unit-cell parameters *a* = *b* = 79.3, *c* = 235.4 Å.

Molecular-replacement calculations with *MOLREP* and *Phaser* (Collaborative Computational Project, Number 4, 1994 ▶) using the structure of a homologous protein [CD155, which has the maximum sequence identity to nectin-1-EC (48.4%) and nectin-2-EC (25.3%); PDB code 3eow; Zhang *et al.*, 2008 ▶] as a search model were unsuccessful. Therefore, heavy-atom derivatives of nectin-1-EC and nectin-2-EC crystals have been prepared for phase determination. Diffraction data sets for the heavy-atom derivatives are currently being collected on BL44XU at the SPring-8 synchrotron facility for phase determination.

## Figures and Tables

**Table 1:** Data-collection statistics Values in parentheses are for the highest resolution shell.

	Human nectin-1α extracellular region	Mouse nectin-2α extracellular region
Beamline	BL44XU, SPring-8	BL44XU, SPring-8
Space group	*P*2_1_3	*P*6_1_22 or *P*6_5_22
Unit-cell parameters (Å)	*a* = *b* = *c* = 164.9	*a* = *b* = 79.3, *c* = 235.4
Wavelength (Å)	0.90000	0.90000
Detector	DIP6040	DIP6040
Crystal-to-detector distance (mm)	540	400
Rotation range per image (°)	0.5	1.0
Total rotation range (°)	60	70
Exposure time per image (s)	20	2
Resolution range (Å)	50.00–2.80 (2.90–2.80)	50.00–2.55 (2.59–2.55)
Total no. of observations	137039 (13666)	61011 (3039)
No. of unique reflections	36109 (3504)	14654 (707)
Completeness (%)	97.9 (96.8)	98.2 (96.8)
〈*I*〉/〈σ(*I*)〉	19.4 (4.3)	21.8 (3.6)
Multiplicity	3.8 (3.9)	4.2 (4.3)
*R*_merge_†	0.068 (0.358)	0.061 (0.396)
Overall *B* factor from Wilson plot (Å^2^)	85.5	58.1

† *R*_merge_ = 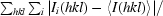 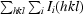, where 〈*I*(*hkl*)〉 is the mean intensity of symmetry-equivalent reflections.
